# Cancer-Cachexia-Induced Human Skeletal Muscle Myotube Degeneration Is Prevented via Cannabinoid Receptor 2 Agonism In Vitro

**DOI:** 10.3390/ph16111580

**Published:** 2023-11-08

**Authors:** John Noone, Mary F. Rooney, Marilena Karavyraki, Andrew Yates, Saoirse E. O’Sullivan, Richard K. Porter

**Affiliations:** 1 School of Biochemistry & Immunology, Trinity College Dublin, D02R590 Dublin, Ireland; john.noone@adventhealth.com (J.N.); marooney@tcd.ie (M.F.R.);; 2 Artelo Bioscience, Ltd., Alderly Edge, Cheshire SK10 4TG, UKsosullivan@artelobio.com (S.E.O.)

**Keywords:** cancer-induced cachexia, myotube degeneration, cannabinoids, ART27.13, CAReS, CB_1_R, CB_2_R

## Abstract

Cachexia syndrome, leading to reduced skeletal muscle and fat mass, is highly prevalent in cancer patients, resulting in further negative implications for these patients. To date, there is no approved therapy for cachexia syndrome. The objective of this study was to establish an in vitro model of cancer cachexia in mature human skeletal muscle myotubes, with the intention of exploiting the cell model to assess potential cachexia therapeutics, specifically cannabinoid related drugs. Having cultured and differentiated primary human muscle myoblasts to mature myotubes, we successfully established two cancer cachexia models using conditioned media (CM) from human colon adenocarcinoma (SW480) and from non-small-cell lung carcinoma (H1299) cultured cells. The cancer-CM-induced extensive myotube degeneration, demonstrated by a significant reduction in mature myotube diameter, which progressed over the period studied. Myotube degeneration is a characteristic feature of cancer cachexia and was used in this study as an index of cachexia. Expression of cannabinoid 1 and 2 receptors (CB_1_R and CB_2_R) was confirmed in the mature human skeletal muscle myotubes. Subsequently, the effect of cannabinoid compounds on this myotube degeneration were assessed. Tetrahydrocannabinol (THC), a partial CB_1_R/CB_2_R agonist, and JWH133, a selective CB_2_R agonist, proved efficacious in protecting mature human myotubes from the deleterious effects of both (SW480 and H1299) cancer cachexia conditions. ART27.13, a full, peripherally selective CB_1_R/CB_2_R agonist, currently being trialled in cancer cachexia (IRAS ID 278450, REC 20/NE/0198), was also significantly protective against myotube degeneration in both (SW480 and H1299) cancer cachexia conditions. Furthermore, the addition of the CB_2_R antagonist AM630, but not the CB_1_R antagonist Rimonabant, abolished the protective effect of ART27.13. In short, we have established a convenient and robust in vitro model of cancer-induced human skeletal muscle cachexia. The data obtained using the model demonstrate the therapeutic potential of ART27.13 in cancer-induced cachexia prevention and provides evidence indicating that this effect is via CB_2_R, and not CB_1_R.

## 1. Introduction

Cachexia syndrome affects >60% of cancer patients and is associated with reduced skeletal muscle and fat mass, reduced tolerance and response to anticancer therapy, reduced physical function, reduced performance status, and reduced quality of life and survival [1]. Indeed, cachexia is estimated to account for 20% of cancer deaths [2]. To date, there is no approved therapy for cachexia syndrome. This study aimed to establish an in vitro model of cancer cachexia in mature human skeletal muscle myotubes, for the purpose of testing potential cachexia therapeutics, specifically cannabinoid related drugs. Previous researchers had already established an in vitro mouse muscle model of cachexia, using C_2_C_12_ myotubes [3,4,5,6]. In this mouse model, addition of conditioned media from mouse colon cancer cells to C_2_C_12_ myotubes induced cachexia. One of the common indices of cachexia observed in this mouse cachexia model was a decrease in myotube diameter [4]. Such models may be used to investigate the mechanisms by which cachexia occurs, thereby improving understanding of the disease and leading to development of further therapeutic strategies. Identified targets and potential therapeutic drugs can be assessed in these models to provide preclinical evidence in advance of, or in conjunction with, in vivo clinical studies. The present study transpired from findings within randomized and nonrandomized clinical trials pointing to the potential benefit cannabinoids may have in the treatment of individuals undergoing palliative care [7]. Literature on the direct effects that such treatments have point to specific benefits to appetite and improvements in body weight in individuals suffering from immunodeficiency virus (HIV) [8]. However, understanding of the direct effect on the skeletal muscle system with cachexia is lacking within this field and requires more attention. Given the integral role skeletal muscle has in physical conditioning and in cancer survival prognosis [9,10], and the evidence to date on the beneficial effects targeting the endocannabinoid system (ECS) has in maintaining muscle function and morphology in mouse models [11], further study into the role such treatments have directly on human skeletal muscle myotubes are required, potentially providing an efficacious treatment for cachexia.

CB_1_ receptors are predominantly found in the central nervous system but have also been detected in the periphery, in adipose tissue, liver cells and skeletal muscle [12,13,14,15]. CB_1_ receptor stimulation has been demonstrated to increase glucose uptake into skeletal muscle [16] and inhibit sarcoplasmic calcium release in skeletal muscle [17]. By contrast, CB_2_ receptors are predominantly expressed in the periphery and have broad tissue and cellular distribution which includes immune cells and skeletal muscle [13,14]. Stimulation of CB_2_ receptors has most commonly been associated with anti-inflammatory responses in immune cells. CB_2_ receptor activation in skeletal muscle reduces ischemia-reperfusion injury [18] and plays a role in myofiber regeneration [19].

**Figure 1:**
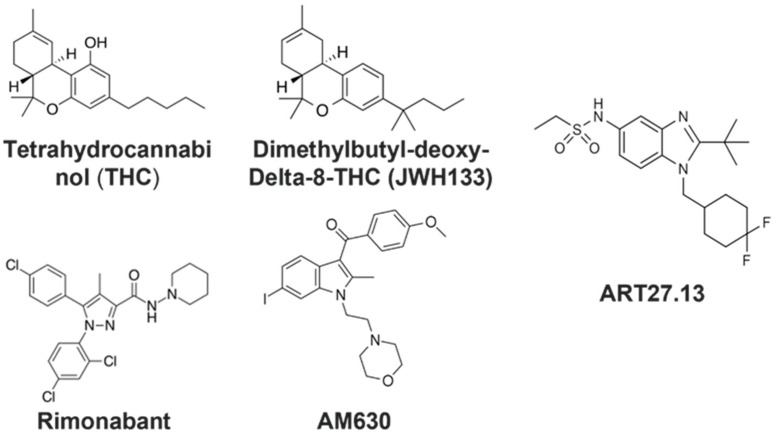
Structures of the cannabinoid receptor agonists/antagonists examined in this study.

Several cannabinoid drugs have emerged as potential therapeutics for various conditions. Specifically, synthetic tetrahydrocannabinol (THC) is licensed in the USA for the treatment of anorexia associated with HIV, and cannabinoid medicines have been used unlicensed or trialled in clinical studies in cancer anorexia/cachexia [20,21,22], anorexia in patients with chronic hepatitis C [23] and in anorexia nervosa [24]. The ability of cannabinoids to modulate body weight and appetite is mediated by both central and peripheral CB_1_ receptors [15]. In this study, we wanted to test a selection of cannabinoid drugs to assess whether they could provide protection against or prevent skeletal muscle cachexia in vitro, in addition to their appetite stimulating effects. The drugs studied (Figure 1) were the partial CB_1_R/CB_2_R agonist THC (Ki CB_1_R ~5 nM; Ki CB_2_R ~3 nM), the selective CB_2_R agonist dimethylbutyl-deoxy-Delta-8-THC (JWH133) (Ki ~3.4 nM), the CB_1_R inverse agonist/antagonist Rimonabant (also known as SR141716, Acomplia, Zimulti), (Ki ~1.6 nM) and the CB_2_R antagonist AM630 (Ki ~30 nM) [25,26]. In addition, we tested the synthetic peripherally selective dual CB_1_R (Ki ~11 nM) and CB_2_R (Ki ~1 nM) agonist ART27.13 (Artelo Biosciences Limited, Shrewsbury, UK) [27,28,29]. Due to ART27.13 being distributed predominately in the periphery, it has shown lower central nervous system side effects than other cannabinoids and is currently in a phase 2 study in cancer patients with weight loss and anorexia assessing whether the drug improves appetites, weight and other measures including quality of life and activity (CAReS; IRAS ID 278450, REC 20/NE/0198) [30]. In the forthcoming results section, we describe the successful establishment of an in vitro model of cancer cachexia and demonstrate the effects of cannabinoids on myotube degeneration in the model.

## 2. Results

### 2.1. In Vitro Human Skeletal Muscle Cancer Cachexia Cell Model Is Established Using Cancer Conditioned Media Which Induces Significant Degeneration of Mature Myotubes

**Figure 2:**
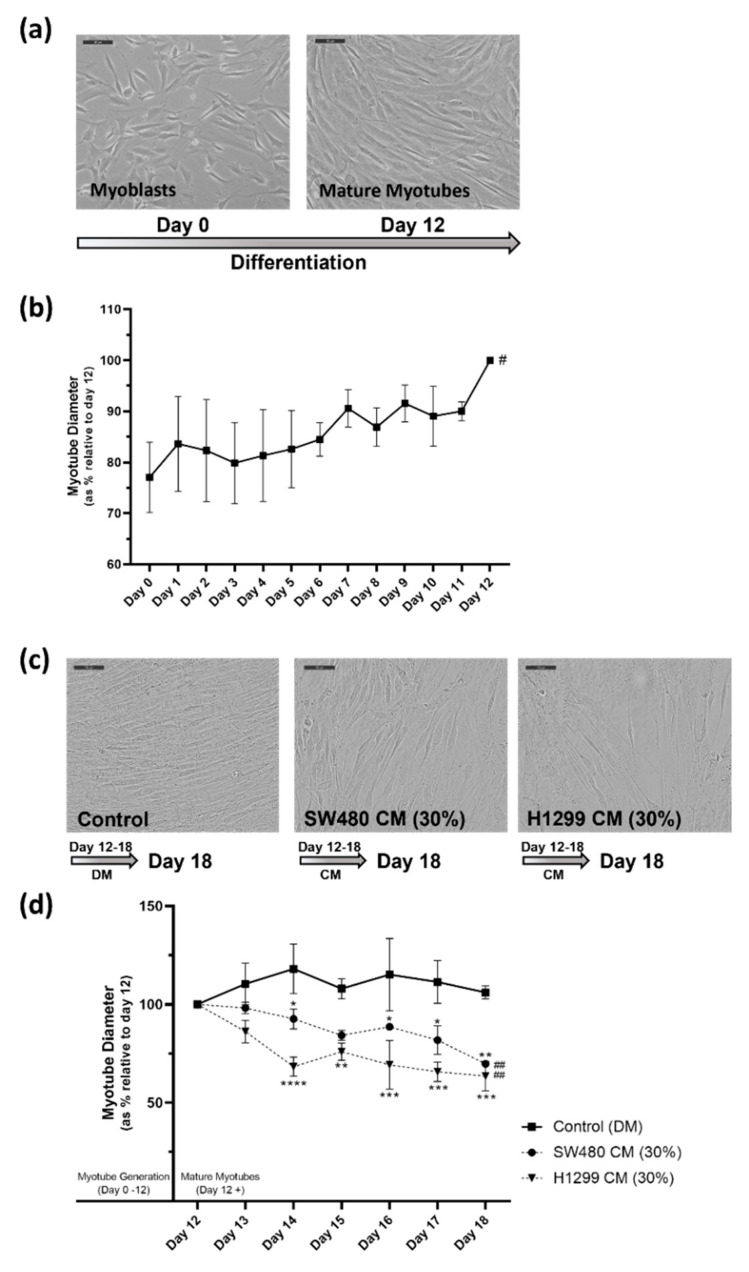
In vitro human skeletal muscle cancer cachexia cell model. (**a**,**b**) Differentiation process (12 days) of myoblasts to myotubes. (**a**) Representative image (20× magnification) of myoblasts (day 0) and mature differentiated myotubes (day 12). Scale bars set to 10 µm. (**b**) Graph demonstrates the increasing myotube diameter from day 0 to day 12, as the myoblasts differentiate to myotubes. Diameters were measured daily during the 12-day differentiation process and myotube diameter data are presented as % relative to diameter of mature myotubes at day 12. (**c**,**d**) At day 12, cancer CM was introduced to induce cachexia; media conditions were, 30% SW480 CM/70% DM, 30% H1299 CM/70% DM or 100% DM for control cells. Cells were monitored and myotube diameters were measured daily for 6 days. (**c**) Representative image (20× magnification) at day 18 for each condition: control (DM), 30% SW480 CM treated and 30% H1299 CM treated myotubes. Scale bars set to 10 µm. (**d**) Graph demonstrates the significant decrease in myotube diameter in the 30% SW480 and 30% H1299 CM cultured cells over the treatment period (# = *p* value ≤ 0.05, ## = *p* value ≤ 0.01) and compared to control myotubes cultured in DM, which maintained quite steady myotube diameters from day 12 to day 18 (* = *p* ≤ 0.05, ** = *p* ≤ 0.01, *** = *p* ≤ 0.001, ****= *p* ≤ 0.0001; compared to control). Data are presented as % relative to diameter of control (DM) mature myotubes at day 12. All data are mean ± SEM of at least *n* = 3.

Primary human skeletal muscle myoblasts (HSkMC) have previously been shown to successfully differentiate into mature myotubes [31,32] with the process taking approximately 10–12 days. Figure 2a,b demonstrates the differentiation process in this study. Figure 2a provides representative images of myoblasts at day 0 and mature differentiated myotubes at day 12, having been grown to confluence and subsequently cultured in a reduced serum media which encourages differentiation, for 12 days. Morphologically, myotube clustering, elongation and stratification is visually noticeable at day 12 when compared to the myoblasts on day 0. Furthermore, Figure 2b displays daily measurements of myotube diameter relative to day 12 diameter, which demonstrates a steady and significant (# = *p* ≤ 0.05) increase in myotube diameter over the 12 days as the myoblasts differentiate into myotubes. Figure S1 provides an example of the myotube diameter measurement procedure in a representative image (Figure S1a) and the data obtained (Figure S1b), which demonstrate in raw myotube diameter values (µm) the progressive increase in myotube diameter throughout differentiation during the 12 days.

The initial objective of this study was to establish an in vitro model of cancer cachexia, in human skeletal muscle. This objective was achieved, and two cancer cachexia models were established, using conditioned media (CM) from human colon adenocarcinoma (SW480) and from non-small-cell lung carcinoma (H1299) cultured cells. The cancer CM was added to mature myotubes at day 12, at a range of concentrations, with the effects monitored for 6 days, up to day 18. The myotubes were examined visually and myotube diameters measured daily, during this period. Both SW480 and H1299 CM significantly impacted the condition of the mature myotubes (Figure 2c). Over the study period, from day 12 to day 18, myotube diameter decreased significantly in the presence of the SW480 CM or H1299 CM. Figure S2 displays the myotube diameter data obtained from the full range of CM concentrations evaluated, for SW480 CM (Figure S2a) and H1299 CM (Figure S2b). Myotubes in standard differentiation media (DM) conditions maintained a relatively consistent myotube diameter over the study period, as did myotubes treated with 10% CM (90% DM) or 20% CM (80% DM). However, a significant reduction in myotube diameter was observed with both SW480 and H1299 CM at 30% CM (70% DM) and 40% CM (60% DM). The higher CM concentrations assessed, e.g., 50% CM (50% DM) and 100% CM, also resulted in decreased myotube diameter, however this effect was observed in just 1 day (at day 13) and thus these higher concentrations were considered too traumatic and not representative of the progressive nature of cachexia seen in vivo.

Considering that the 30% CM condition induced a steady and progressive decrease in myotube diameter, that was significantly different from that observed in myotubes cultured in standard DM conditions, which maintained their size, with no significant change in diameter from day 12 to day 18, the 30% CM (70% DM) condition was chosen for both the SW480 and the H1299 cancer cachexia in vitro cell models. The use of such a concentration is also supported based on previous studies within the field, with both Jackman et al. (2017) and Seto et al. (2015) using concentrations of 33% conditioned media in their induction and assessment of cachexia in C_2_C_12_ myotubes in vitro [3,4]. Figure 2d demonstrates the significant (*## = p* ≤ 0.01) reduction in myotube diameter from day 12 to day 18 in the presence of either 30% SW480 CM or 30% H1299 CM, and the reduction versus the control (DM) myotubes (at day 18, versus DM; 30% SW480 CM, ** = p ≤ 0.01 and 30% H1299 *** = *p* ≤ 0.001). The progressive decrease in mature myotube diameter is considered myotube degeneration, and this is the terminology used in this study to describe this effect. Myotube degeneration is a characteristic feature of cachexia and is used in this study as our phenotypic measurement of cachexia.

### 2.2. Cannabinoid 1 and 2 Receptors Are Expressed in the HSkMC Used in this Study

**Figure 3:**
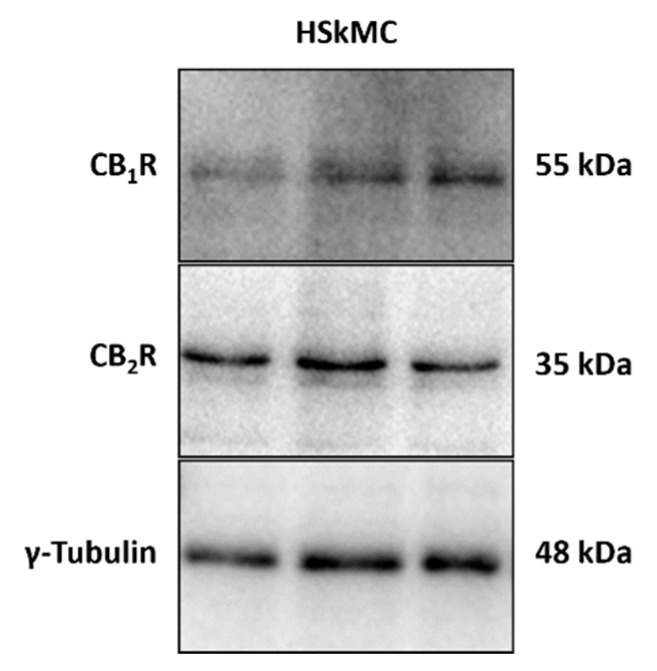
Cannabinoid receptors type 1 and 2 expression in HSkMC. Immunoblot of mature differentiated myotubes (untreated) at day 12 confirmed expression of both cannabinoid receptor type 1 (CB_1_R, 55 kDa) and cannabinoid receptor type 2 (CB_2_R, 35 kDa) proteins in the human skeletal muscle cell line used in the present study. Tubulin expression (γ-tubulin, 48 kDa) was also assessed, as a control. Cell samples were collected independently, and three of these replicate samples were prepared and assessed simultaneously on the immunoblot shown. Antibody details are provided in methods Section 4.5. Uncropped images are provided in Figure S3.

Expression of the cannabinoid receptors type 1 and 2 (CB_1_R and CB_2_R) has previously been detected in several mammalian skeletal muscle tissues and cultured cells, at the mRNA and protein level [15,17,33,34]. More pertinent to the present study, CB_1_R and CB_2_R mRNA and protein expression has been detected in human skeletal muscle tissue homogenates and cultured primary cells [12,13,35,36]. In the interest of rigor, we assessed via immunoblotting whether CB_1_R and CB_2_R were expressed in these mature HSkMC myotubes (at day 12, control (DM) conditions). As displayed in Figure 3, we confirmed that both CB_1_R and CB_2_R proteins are expressed in these cells.

### 2.3. Cannabinoid Receptor Agonists, THC and JWH133, Provide a Protective Effect against Myotube Degeneration in the Cancer Cachexia Model, with SW480 and H1299 Cancers

Having established the in vitro human skeletal muscle cell model of cancer cachexia, our next objective was to exploit this model to test whether a selection of cannabinoid related drugs had any protective effects against myotube degeneration.

**Figure 4:**
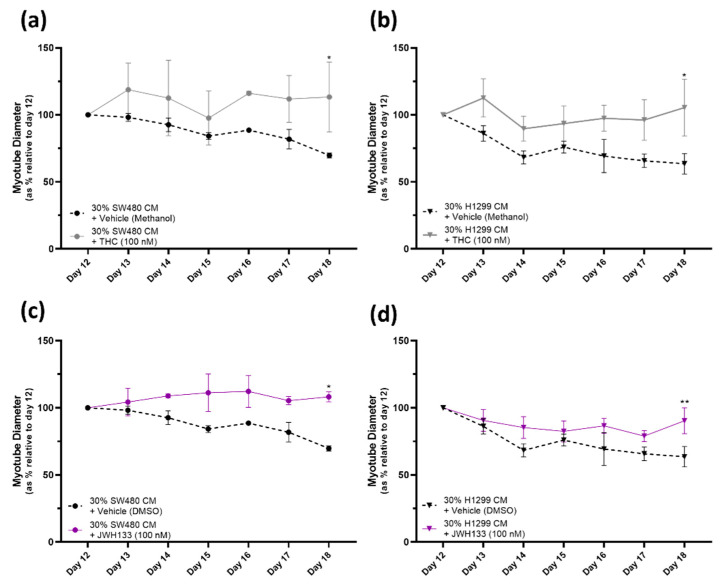
Protective effect of CB_1/2_ receptor agonist THC and selective CB_2_R agonist JWH133 treatment against myotube degeneration due to SW480/H1299-CM-induced cachexia. (**a**–**d**) At day 12, cancer CM was introduced to induce cachexia; 30% SW480 CM/70% DM or 30% H1299 CM/70% DM and, simultaneously, THC [100 nM], JWH133 [100 nM] or vehicle (Methanol/DMSO [0.1%]) was added. Cells were monitored and myotube diameters were measured daily for 6 days. Data are presented as % relative to diameter of mature myotubes at day 12. THC and JWH133 treatment groups were compared to their vehicle treated counterparts (* = *p* ≤ 0.05, ** = *p* ≤ 0.01). All data are mean ± SEM of at least *n* = 3. (**a**,**b**) Data demonstrate that THC [100 nM] protects against myotube degeneration in both SW480- (**a**) and H1299- (**b**) CM-induced cachexia; it prevents the reduction in myotube diameter observed in the vehicle- (methanol [0.1%]) treated-cancer-CM-induced cachexia groups. (**c**,**d**) Data demonstrate that JWH133 [100 nM] protects against myotube degeneration in both SW480- (**c**) and H1299- (**d**) CM-induced cachexia; it prevents the reduction in myotube diameter observed in the vehicle- (DMSO [0.1%]) treated-cancer-CM-induced cachexia groups.

Initially, two cannabinoid receptor agonists were assessed: the partial CB_1_R/CB_2_R agonist tetrahydrocannabinol (THC) and the selective CB_2_R agonist dimethylbutyl-deoxy-Delta-8-THC (JWH133). Both THC and JWH133 provided a protective effect against myotube degeneration due to both SW480- and H1299-CM-induced cachexia. Figure 4a shows that the THC [100 nM] treatment had a significantly (at day 18, THC versus vehicle, *p* ≤ 0.05) protective effect against the 30% SW480 CM cancer cachexia condition. THC treatment also prevents the myotube diameter reduction resulting from the 30% H1299 CM cancer cachexia model (at day 18, THC versus vehicle, * = *p* ≤ 0.05) (Figure 4b).

A similarly protective effect is observed with the selective CB_2_ receptor agonist JWH133 [100 nM] in both 30% SW480 and 30% H1299 CM cancer cachexia conditions. The myotube degeneration induced via the cancer cachexia conditions observed with the vehicle treatment is significantly reduced in the JWH133 treated groups (JWH133 [100 nM] versus vehicle at day 18: 30% SW480 CM, * = p ≤ 0.05 and 30% H1299 CM, ** = p ≤ 0.01). Endocannabinoid *N*-arachidonoylethanolamine (AEA), a CB_1_R/partial CB_2_R agonist, was assessed in the same manner. Treatment with AEA [100 nM] did not affect the myotube degeneration caused by SW480- or H1299-CM-induced cachexia (Figure S4).

### 2.4. CB_1_R Antagonist Rimonabant or CB_2_R Antagonist AM630 Do Not Affect the Myotube Degeneration Induced by Either the SW480 or H1299 Cancer Cachexia Model

**Figure 5:**
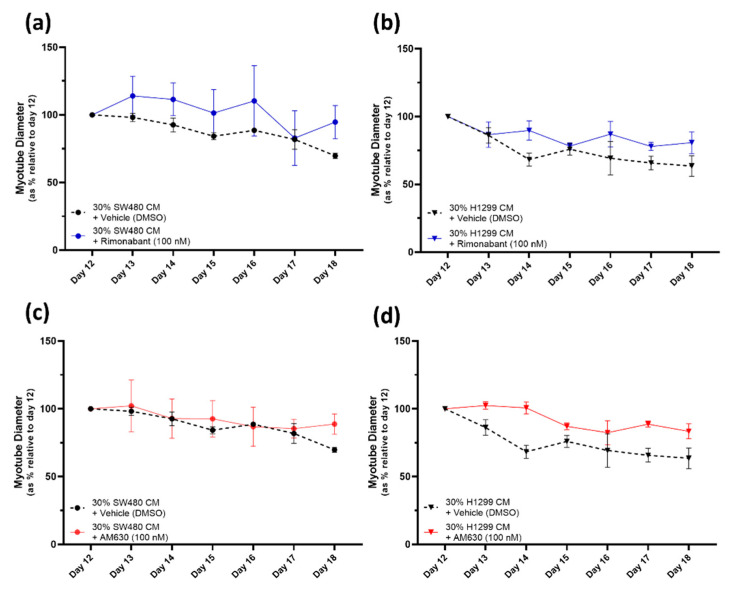
Treatment with CB_1_R antagonist Rimonabant or CB_2_R antagonist AM630 does not affect the myotube degeneration caused by SW480/H1299-CM-induced cachexia. (**a**–**d**) At day 12, cancer CM was introduced to induce cachexia; 30% SW480 CM/70% DM or 30% H1299 CM/70% DM and, simultaneously, Rimonabant [100 nM], AM630 [100 nM] or vehicle (DMSO [0.1%]) was added. Cells were monitored and myotube diameters were measured daily for 6 days. Data are presented as % relative to diameter of mature myotubes at day 12. All data are mean ± SEM of at least *n* = 3. Rimonabant and AM630 treatment groups were compared to their vehicle-treated counterparts. (**a**,**b**) Data demonstrate that Rimonabant [100 nM] does not protect against the myotube degeneration caused by either the 30% SW480- (**a**) or the 30% H1299- (**b**) CM-induced cachexia. Rimonabant treated groups display the same gradual decrease in myotube diameter over time as the vehicle control groups. (**c**,**d**) Data similarly demonstrate that AM630 [100 nM] does not protect against the myotube degeneration caused by either the 30% SW480- (**c**) or the 30% H1299- (**d**) CM-induced cachexia. AM630 treated groups display the same gradual decrease in myotube diameter over time as the vehicle control groups.

Next, we assessed two cannabinoid receptor antagonists: the inverse agonist/antagonist of CB_1_ receptor, Rimonabant, and AM630, a selective antagonist/inverse agonist for the CB_2_ receptor. Neither Rimonabant [100 nM] nor AM630 [100 nM] affected the myotube degeneration induced by either cancer CM (Figure 5a,b and Figure 5c,d, respectively).

### 2.5. ART27.13 Protects against Cancer-Induced Skeletal Muscle Cachexia via Agonism of the Cannabinoid Receptor Type 2

**Figure 6:**
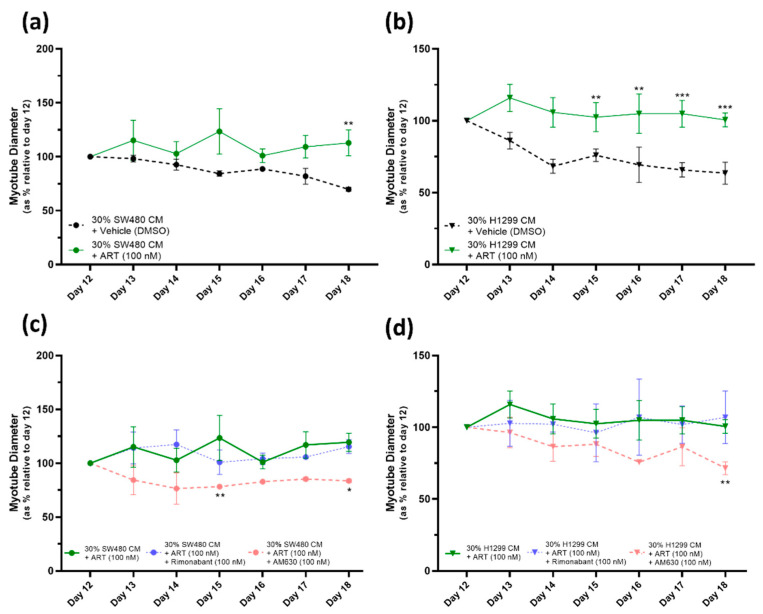
ART27.13 protects against cancer-induced skeletal muscle cachexia via agonism of the cannabinoid receptor type 2. (**a**–**d**) At day 12, cancer CM was introduced to induce cachexia: 30% SW480 CM/70% DM or 30% H1299 CM/70% DM. Drug treatments were added simultaneously. Cells were monitored and myotube diameters were measured daily for 6 days. Data are presented as % relative to diameter of mature myotubes at day 12. All data are mean ± SEM of at least *n* = 3. (**a**,**b**) Data demonstrate that ART27.13 prevents the myotube degeneration caused by both SW480- (**a**) and H1299- (**b**) CM-induced cachexia. The vehicle (DMSO [0.1%]) has no impact on the myotube degeneration caused by the cancer-CM-induced cachexia, a gradual decrease in myotube diameter is observed. Whereas the ART27.13 [100 nM] treated myotubes, subjected to the same cancer CM cachexia conditions, do not display myotube degeneration, with myotube diameter remaining steady over the treatment period. Significant differences between ART27.13 and vehicle treated groups are shown (* = *p* ≤ 0.05, ** = *p* ≤ 0.01 and *** = *p* ≤ 0.001). (**c**,**d**) Graphs include the 30% SW480 CM + ART27.13 [100 nM] (**c**) and the 30% H1299 CM + ART27.13 [100 nM] (**d**) data group as per (**a**) and (**b**), respectively, demonstrating the protective effect of ART27.13 against myotube degeneration. The two further treatment groups assess whether the addition of CB_1_R selective antagonist Rimonabant and/or CB_2_R selective antagonist AM630 interfere with the beneficial effect of ART27.13. In both 30% SW480- (**c**) and 30% H1299- (**d**) cancer-CM-induced cachexia conditions, the CB_1_R antagonist Rimonabant does not affect the protection provided by ART27.13. However, the CB_2_R antagonist AM630 significantly negates the protection against myotube degeneration provided by ART27.13 treatment. Significant differences between combined ART27.13 and antagonist treatment versus ART27.13 treatment alone are shown (* = *p* ≤ 0.05, ** = *p* ≤ 0.01).

We next tested the Artelo Biosciences drug ART27.13, a synthetic peripherally selective cannabinoid receptor agonist, which has previously been shown to have an affinity for both CB_1_R and CB_2_R, albeit at a higher affinity for CB_2_R. Our studies into the efficacy of ART27.13 [100 nM] in combination with 30% CM illustrates that ART27.13 [100 nM] provides significant (at day 18 ART27.13 treatment versus vehicle, in 30% SW480 CM model, ** = *p* ≤ 0.01 and in 30% H1299 CM, *** = p ≤ 0.001) protection against the deleterious effects of both cancer CMs (Figure 6a,b). The cancer cachexia cell models induce a gradual, but significant, decrease in myotube diameter from day 12 to day 18. The vehicle (DMSO [0.1%]) has no impact on this myotube degeneration caused by the SW480- or H1299-CM-induced cachexia. However, as Figure 6a,b show, the ART27.13 [100 nM] treated myotubes, subjected to the same cancer CM cachexia conditions, do not display myotube degeneration during the study period, with myotube diameter remaining steady from day 12 to day 18.

Furthermore, addition of the CB_1_R antagonist Rimonabant [100 nM] to ART27.13 treated myotubes in the cancer cachexia model had no impact on the protective affect against myotube degeneration provided by ART27.13 [100 nM], in either the SW480 or H1299 cancer cachexia cell model (Figure 6c and 6d, respectively). However, the addition of the CB_2_R antagonist AM630 [100 nM] abolished the protective effect of ART27.13 [100 nM] against both SW480- and H1299-cancer-induced cachexia.

## 3. Discussion

The object of this study was to establish an in vitro model of cachexia in mature HSkMC myotubes with view to testing for anti-cachexia drugs. Transcriptome and morphological analyses have established that it takes 12 days to produce fully differentiated HSkMC myotubes [31]. It was observed in this study that the predicted morphological changes in myotubes were manifest, during the course of differentiation, and were commensurate with a significant increase in average diameter.

Cachexia is commonly associated with human lung and gastrointestinal cancer [2,37,38,39]. Hence, we predicted that conditioned media from cultured lung and colon cells would be likely to induce cachexia in cultured human skeletal muscle cells (myotubes). It was established that conditioned media from human non-small lung cancer cells (H1299) and human colon cancer (SW480), at a concentration of 30%, resulted in significant reduction in human myotube diameter (of ~2.5–3 µm (~35–45% relative to day 12) over six days (day 12–18). We speculate that these adverse effects may be driven by known skeletal muscle atrophy protagonists such as Interleukin-6 (IL-6), IL-8, Heat shock protein 70 (Hsp70) and Hsp90, respectively, all of which are known to be highly expressed and secreted from H1299 and SW480 cell lines according to the Cancer Cell Line Encyclopedia (CCLE) database (https://depmap.org/portal/ccle/ (accessed on 29 October 2023)). However, more work is needed to support this conclusion. Thus, the significant reduction in human myotube diameter was used as an index of cachexia in this model.

The use of condition media, with a subsequent reduction in diameter of myotubes, as an index of cachexia, has previously been established in C_2_C_12_ mouse myotubes [3,4]. Equivalent changes in myotube diameter were observed in this study when compared to the C_2_C_12_ myotube model, where, for example, condition media from mouse C26 colon carcinoma cells resulted in a 15–20% reduction in C_2_C_12_ myotube diameter [4].

Having established a HSkMC in vitro model of cachexia it was hypothesised that cannabinoid receptors on skeletal muscle might have a direct role in protecting against cachexia. Previous researchers have already established that CB_1_R and CB_2_R are present in human skeletal muscle [13,33,40], and in this study, expression of both receptions was confirmed in mature HSkMC cultured over the 12 days. Subsequently, efficacy of selected cannabinoid compounds was tested in the model. The fact that the prototypical cannabinoid, tetrahydrocannabinol (THC), a partial CB_1_R/CB_2_R agonist, proved efficacious in preventing reduction in myotube diameter indicated that either the CB_1_ receptor and/or the CB_2_ receptor mediated this effect. The selective CB_2_R agonist dimethylbutyl-deoxy-Delta-8-THC (JWH133) also proved efficacious, suggesting the CB_2_ receptor is mediating the anti-cachexia effect.

The CB_1_ receptor has been shown to be involved in the regulation of mitochondrial ultrastructure, oxidative metabolism and insulin sensitivity impacting skeletal muscle growth, force and fatigability [11,41,42,43]. Whereas, the CB_2_ receptor has been shown to have a specific role in skeletal muscle regeneration and metabolism, potentially driven from antioxidative and inflammatory processes [18,19,43]. However, this is the first evidence to suggest that targeting the endocannabinoid receptors may directly maintain HSkMC phenotype under cachexic conditions in vitro.

By contrast, the CB_1_R agonist Rimonabant (also known as SR141716, Acomplia, Zimulti) and the selective CB_2_R antagonist AM630 could not counteract the deleterious effect of conditioned media in reducing myotube diameter, indicating that these CB_1_R or CB_2_R antagonists alone cannot counteract the cachexic nature of the conditioned media.

The Artelo plc drug ART27.13 is a synthetic peripherally selective full CB_1_R (Ki ~11 nM)/CB_2_R (Ki ~1 nM) agonist undergoing Phase 2 clinical trials in cancer patients with weight loss and anorexia. Since the initial data suggested cannabinoid receptor activation can counteract the deleterious effects of cancer CM on myotube diameter, we tested ART27.13 in our model. ART27.13 was effective at protecting mature HSkMC myotubes against the deleterious effect induced by CM from both SW480 and H1299 cancers. Theoretically, this drug, at the concentration used [100 nM], could have acted via either the CB_1_R or CB_2_R and it is believed that the CB_1_ effects primarily drive positive changes in appetite, weight gain and metabolism. To establish whether either or both receptors were involved in preventing reduction in myotube diameter, ART27.13 was tested in combination with cannabinoid receptor antagonists. The fact that CB_1_ receptor antagonism (using Rimonabant) does not interfere with the protective effects of ART27.13 suggests ART27.13 is not acting via the CB_1_R. By contrast, the addition of a CB_2_ receptor antagonist (AM630) inhibited the ability of ART27.13 to protect against the deleterious effects of cancer CM on human myotubes, indicating that ART27.13 acts via the CB_2_R.

Taken together, the evidence indicates that ART27.13 and other CB_2_R agonists, such as THC and JWH133, are directly efficacious against human muscle cachexia and may act to maintain muscle composition from an in vivo perspective. Given the correlation between an increase in cachexia and reduction in patient quality of life [7,8], maintenance of skeletal muscle mass in the presence of cachexic conditions may not only improve mobility but further act to improve cancer patient survival. This preclinical observation bodes well for the current clinical trial with ART27.13 on patients with cachexia (ISRCTN15607817 also known as CAReS study). In addition, it is clear the protective effect of ART27.13 is via the CB_2_ receptor and not the CB_1_ receptor; as such, targeting the CB_2_R receptor may provide further benefits to these patients. However, more investigation is needed to fully elucidate mechanisms of action.

The efficacy of THC, JWH133 and ART27.13 in protecting myotube diameter in this in vitro cachexia model provides a new role for CB_2_R agonism. The CB_2_R is often associated with inflammatory tissues and is thought to be responsible for the anti-inflammatory actions of endogenous and exogenous cannabinoids [44]. Future work will determine the mechanism by which CB_2_R agonists protect against cachexia in this model. However, this preclinical evidence strongly supports a role for CB_2_ agonism in the maintenance of HSkMC diameter under cachexic conditions in vitro.

## 4. Materials and Methods

### 4.1. Primary Human Skeletal Muscle Cell (HSkMC) Culture and Differentiation

#### 4.1.1. Human Skeletal Muscle Cell Culture

Primary human skeletal muscle cells (HSkMC) (ATCC, Manassas, VA, USA; #PCS-950-010) were cultured as described by Noone et al. (2023) [31]. Briefly, HSkMC myoblasts were maintained in the mid-exponential growth phase at 37 °C in a humidified environment with 5% CO_2_. Cells were grown in Dulbecco’s Modified Eagle’s Medium (DMEM), high glucose, GlutaMAX^TM^ (Gibco, Waltham, MA, USA; cat# 10569010) media supplemented with 10% Fetal Bovine Serum (FBS) (Gibco, Waltham, MA, USA; cat# 302025) and 2% penicillin-streptomycin (Gibco, Waltham, MA, USA; cat# 15070063) (hereafter; GM). Sterile techniques were employed during all cell culture procedures and standard sterile single use plasticware (obtained from Cruinn Ltd., Dublin, Ireland, unless stated otherwise) was used. Routine testing for mycoplasma infections was performed as per the method described by Young et al. (2010) [45]. HSkMC were passaged at 70–80% confluence; the adherent myoblasts were detached using TrypLE express dissociation reagent (Gibco, Waltham, MA, USA cat# 12605028) and sub-cultured as appropriate for continued growth and seeded for differentiation as described in Section 4.1.2. Prior to seeding myoblasts for differentiation, cell viability was assessed using the trypan blue technique, to ensure seeding densities were accurate and consistent.

#### 4.1.2. Differentiation of Human Skeletal Muscle Myoblasts to Mature Myotubes

HSkMC myoblasts were seeded at 5 × 10^4^ cells/well in 24-well plates and cultured as described above. Once the HSkMC reached 80–90% confluence (after approximately 4–5 days), differentiation was induced by removing the existing growth media, and replacing it with differentiation media (DM) which contained a reduced serum concentration. DM was composed of DMEM, high glucose, GlutaMAX^TM^ (Gibco, Waltham, MA, USA cat# 10569010) supplemented with 2% Horse Serum (HS) (Gibco, Waltham, MA, USA; 16050130) and 2% penicillin–streptomycin (Gibco, Waltham, MA, USA; cat# 15070063). Myotube differentiation was monitored by regular visual inspection using a Leica DM1L light microscope and DM was replenished at regular intervals. Differentiated myotubes were deemed mature myotubes after day 10–12 based on previous evidence described in Noone et al. (2023) [31] and Levitt et al. (2019) [32].

### 4.2. Measurement of Myotube Diameter

Measurement of myotube diameter was performed as per the methodology described by Jackman et al. (2017) [3]. Phase micrographs were taken at regular intervals at magnification 10× using the live-cell imaging and analysis platform IncuCyte ^®^S3 system. The same imaging and measurement procedures were employed for all experimental groups throughout this study. To ensure consistency in confluence within the well, four images of each well (left, right, top and bottom) in a 24-well plate were taken per time point and ImageJ (version 1.53t) imaging software was used to measure myotube diameters. The diameter (µM) of each myotube was an average of 3 measurements along the myotube, with the same 12 myotubes assessed per time point per group for 6 days (216 measures in total per group). Figure S1 provides an example of the measurement procedure (Figure S1a) and the data obtained (Figure S1b). The ImageJ measurement data were coupled to Excel spreadsheets; % change in diameter (µM) relative to day 12 was calculated and the data were graphed using GraphPad Prism software (version 9). For measuring myotube diameter, a 10× magnification was used, which provided a large reading frame, allowing myotubes to be measured. However, the 10× magnification images are not ideal for visual presentation in an article therefore where representative images are presented in the article, 20× magnification images (of the same cells) have been used, which provide clearer visibility of the cells in an article format.

### 4.3. In Vitro Cancer Cachexia Model

#### 4.3.1. Conditioned Media from Cancer Cell Lines

Conditioned media from cancer cell lines was used to induce cachexia in our model of in vitro cancer cachexia. Conditioned media was harvested from two cancer cell lines: a human epithelial non-small-cell lung cancer, H1299 cells (Gifted by Parviz Motlagh laboratory, Umeå University, Umeå, Sweden), and a human epithelial colorectal adenocarcinoma line, SW480 (ATCC, Manassas, VA, USA; cat# CCL-228). H1299 and SW480 cancer cells were grown in DMEM, high glucose, GlutaMAX^TM^ (Gibco, Waltham, MA, USA cat# 10569010) supplemented with 10% FBS (Gibco, Waltham, MA, USA cat# 302025) and 2% penicillin–streptomycin (Gibco, Waltham, MA, USA cat# 15070063). Cells were cultured until confluent, at which point, H1299 and SW480 cells were sub-cultured in DM (described in Section 4.1.2), to mimic the conditions used to differentiate the HSkMC myoblasts to myotubes. H1299 and SW480 cells were subsequently grown to confluency and the ‘conditioned media (CM)’ was collected. CM was aspirated, collected and centrifuged at 600× *g* for 5 min to remove cell debris. CM was aliquoted and stored at −80 °C until required.

#### 4.3.2. Culture of Mature Myotubes with Cancer Conditioned Media

HSkMC myoblasts were cultured, seeded and differentiated into myotubes as described in Section 4.1. On day 12, the mature myotube culture conditions were altered to mimic cancer conditions and induce cancer cachexia by replacing the DM with either a 30% H1299 CM/70% DM mixture or a 30% SW480 CM/70% DM mixture. Control cells were provided fresh DM. Cells were monitored closely and imaged as per Section 4.2, to measure myotube diameter. Change in myotube diameter over the study period was used as an index of the myotube condition. Reduced myotube diameter compared to control (DM) indicated myotube degeneration was occurring, as seen in cachexia [4], due to the cancer CM.

Throughout this study, and hence for all data presented here within, the cancer cachexia conditions applied were 30% CM/70% DM, for both H1299 CM and SW480 CM. This concentration was selected based on the results obtained from an initial assessment of the effects of a range of concentrations of CM on mature myotubes (Figure S2).

### 4.4. Treatment of Myotubes with Cannabinoid Receptor Agonists/Antagonists

As previously stated, HSkMC myoblasts were cultured, seeded and differentiated into myotubes as described in Section 4.1. Mature myotubes were treated with a selection of CB_1_R and CB_2_R agonists and antagonists, on day 12, in combination with the introduction of the CM, to assess their potential effectiveness in mitigating against the myotube degeneration due to the 30% H1299 or SW480 CM, compared to the vehicle control. The cells were monitored for 6 days and imaged for myotube diameter measurements, as described previously. The drugs [final concentration] used in this study were as follows: THC [100 nM] (Merck, Boston, MA, USA; cat# T4764), JWH133 [100 nM] (Bio-techne, Minneapolis, MN, USA; cat# 1343), AEA [100 nM] (Merck, St. Louis, MO, USA; cat# A0580) (Supplementary Materials), Rimonabant [100 nM] (Merck, St. Louis, MO, USA; cat# SML0800), AM630 [100 nM] (Merck, St. Louis, MO, USA; cat# SML0327) and ART27.13 [100 nM] (supplied by Artelo Biosciences, formerly AZD1940 [15]). Possession and use of THC was granted under Department of Health (Ireland) licence number 5/549-1-2020. The chemical structures are provided in Figure 1, in the introduction section. Drugs were prepared fresh in the appropriate culture media, from concentrated stocks prepared in methanol (THC and AEA) or DMSO (JWH133, Rimonabant, AM630 and ART27.13). The final concentration of vehicle in the treatment well was 0.1%, accordingly, for the vehicle control wells; methanol or DMSO was added to a final concentration of 0.1%. Figure S5 demonstrates that the vehicle treatments have no effect on the mature myotubes in either control DM or cancer cachexia conditions and Figure S6 shows that these drugs had no effect on myotube diameter under standard DM (control) conditions.

### 4.5. Immunoblotting (Cannabinoid Receptor 1 and 2 Expression)

Immunoblotting was performed to detect expression of the CB_1_ and CB_2_ receptors in the HSkMC used in this study. All reagents used were purchased from Merck Group, St. Louis, MO, USA, unless stated otherwise. Cell culture and differentiation procedures were carried out as described in Section 4.1. At day 12, mature myotubes were collected, pelleted with centrifugation for 10 min at 600× *g* and stored at −80 °C. To prepare samples for immunoblotting, cell pellets were thawed on ice, and cells were lysed to release cellular proteins. Protein concentration was determined using the Bicinchoninic Acid (BCA) Assay as described by Smith et al. (1985) [46], to enable equal amount of protein to be added to each well. Using the methods originally described by Laemmli (1970) [47], proteins were resolved with sodium dodecyl sulphate-polyacrylamide gel electrophoresis (SDS-PAGE) and transferred to polyvinylidene difluoride (PVDF) membranes (Immobolin-PSQ; Merck, St. Louis, MO, USA; cat# IPVH00010) using a semi-dry transfer system (Hoefer Inc., Holliston, MA, USA). Membranes were blocked via incubation in tris-buffered saline (20 mM tris-HCl, 150 mM NaCl, pH 7.6) with 0.1% (*v*/*v*) Tween (TBST), supplemented with 5% (*w*/*v*) non-fat dry milk powder for 1 h at room temperature. Blots were then incubated with primary antibodies diluted in fresh blocking buffer overnight at 4 °C, with CB_1_R rabbit polyclonal antibody (ABM, Glasgow, UK, cat# Y080037) at a 1:500 dilution and the CB_2_R mouse monoclonal antibody (Sigma-Aldrich/Merck, St. Louis, MO, USA, cat# WH0001269M1) at 1:1000. Although CB_2_R was originally identified based on its similarity to CB_1_R, the protein sequence homology is less than 50% and the proteins differ in size; the 472 amino acid CB_1_R protein resolves at 55 kDA, while the considerably smaller 360 amino acid CB_2_R protein is detected at 35 kDa. γ-Tubulin (Novus Biologicals, Littleton, CO, USA, cat# NB110-90616) was used as a loading control. Following primary antibody incubation overnight, membrane was incubated with horseradish-peroxidase-linked (HRP) secondary antibody (Promega, Madison, WI, USA; anti-rabbit HRP conjugated antibody, cat# W4011; anti-mouse HRP conjugated antibody, cat# W4021) at 1:1000 dilution in a blocking buffer, for 1 h at room temperature. Proteins were detected using an enhanced chemiluminescence (ECL) substrate, detecting horseradish-peroxidase-labelled antibody, by means of the HRP catalysed oxidation of luminol under alkaline conditions and the results were visualised with a ChemiDoc (Bio-Rad, Hercules, CA, USA) computerised system with the accompanying Image Lab software (version 1.53t).

### 4.6. Statistical Analysis

Statistical analysis and graph preparation was performed using GraphPad Prism software (version 9). Data are presented as the mean ± standard error of the mean (SEM), of at least three independent experiments. Mean values were compared using a two-way (time × treatment) repeated measures analysis of variance (ANOVA), with a Bonferroni multiple comparison post-test to measure differences between treatment and control groups. *P* values, where shown, indicate significance over time (#) or between groups (*) (*/# = *p* ≤ 0.05, **/## = *p* ≤ 0.01 and ***/### = *p* ≤ 0.001).

## 5. Conclusions

In vitro treatment of HSkMC myotubes with 30% human colon adenocarcinoma (SW480) and non-small-cell lung carcinoma (H1299) conditioned media significantly reduces HSkMC myotube diameter. Cannabinoid receptor agonists, THC, JWH133 and ART27.13 provide a protective effect against this HSkMC myotube degeneration via CB_2_R agonism in vitro. This evidence supports a role for targeting CB_2_R agonism in cancer cachexia in vivo.

## Data Availability

The data presented in this study are contained within the article and Supplementary Material. Request for further details can be directed to corresponding author.

## Supplementary Materials

The following supporting information can be downloaded at: https://www.mdpi.com/article/10.3390/ph16111580/s1, Figure S1: Measurement of myotube diameter, with increasing diameter as an indicator of differentiating myotubes. Figure S2: Optimization of conditioned media concentration for in vitro human skeletal muscle cancer cachexia cell model. Figure S3: Uncropped cannabinoid receptors type 1 and 2 immunoblots. Figure S4: Effect of the endocannabinoid *N*-arachidonoylethanolamine (AEA) (CB_1_R/partial CB_2_R agonist) on cachexia model. Figure S5: Effect of vehicles on myotubes. Figure S6: Effect of cannabinoid drugs on control (DM) myotubes.
