# Supplementary material for: Cancer-Cachexia-Induced Human Skeletal Muscle Myotube Degeneration Is Prevented via Cannabinoid Receptor 2 Agonism In Vitro
Source: Pharmaceuticals (Basel). 2023 Nov 8;16(11):1580. doi: 10.3390/ph16111580 (PMC10675367; doi:10.3390/ph16111580)
Supplement: Supplementary file 1 [file pharmaceuticals-16-01580-s001.zip › pharmaceuticals-2689186-supplementary.pdf]

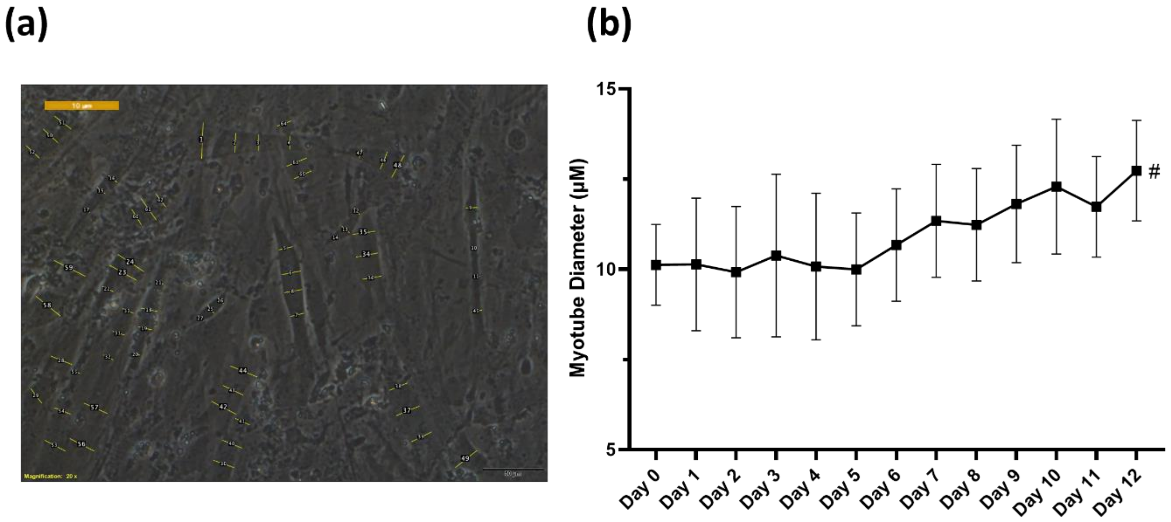

Figure S1. Measurement of myotube diameter, with increasing diameter as an indicator of differentiating myotubes. (a) Representative image (20X magnification) of myotube diameter measurements. **(b)** Graph of raw myotube diameter measurements (μM) demonstrates a significant increase in myotube diameter over the 12-day differentiation period (# = *p* value ≤ 0.05) as the myoblasts differentiate into myotubes.

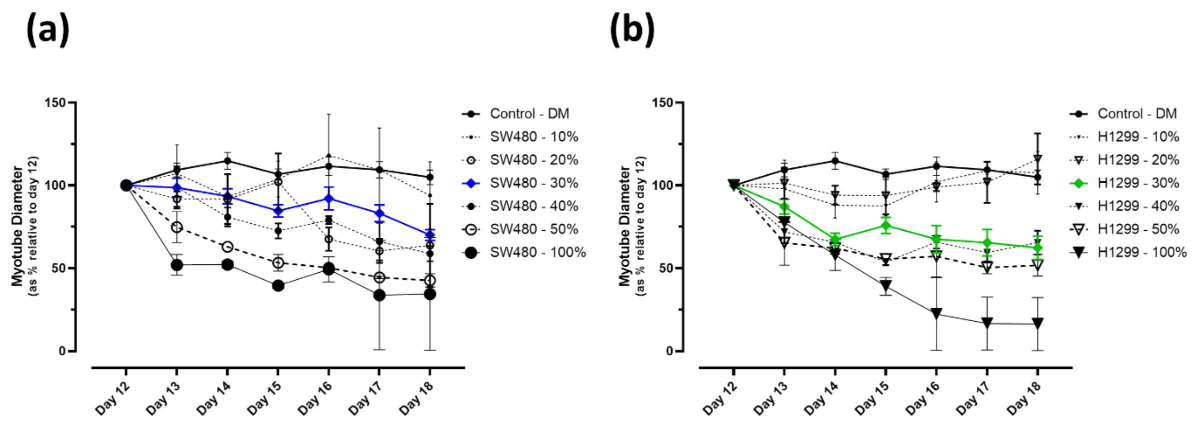

Figure S2. Optimization of conditioned media concentration for *in-vitro* human skeletal muscle cancer-cachexia cell model. A range of CM concentrations were introduced to the mature myotubes at day 12 and cells were monitored and myotube diameter was measured daily for 6 days. Data is presented as % relative to diameter of mature myotubes at day 12. Increasing concentrations of cancer conditioned media resulted in increased myotube degeneration (as measured by decreased myotube diameter). **(a)** Assessment of SW480 CM concentrations; control (100% DM), 10% SW480 CM (90% DM), 20% SW480 CM (80% DM), 30% SW480 CM (70% DM), 40% SW480 CM (60% DM), 50% SW480 CM (50% DM) and 100% SW480 CM. **(b)** Assessment of H1299 CM concentrations; control (100% DM), 10% H1299 CM (90% DM), 20% H1299 CM (80% DM), 30% H1299 CM (70% DM), 40% H1299 CM (60% DM), 50% H1299 CM (50% DM) and 100% H1299 CM.

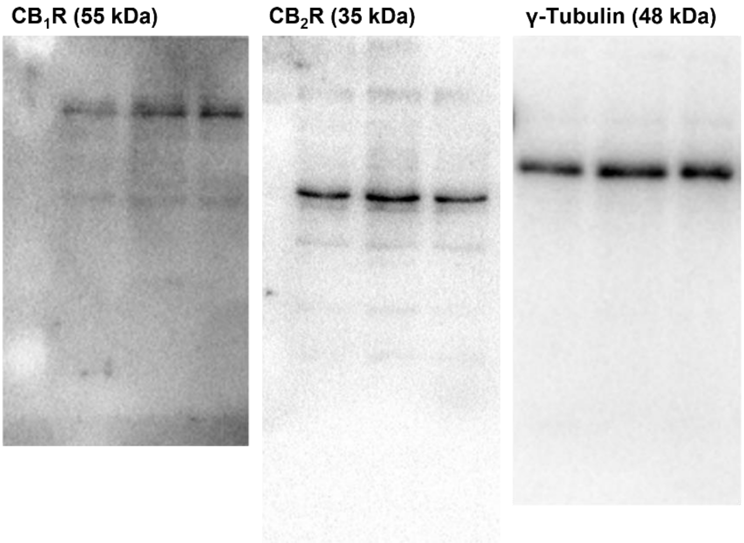

Figure S3. Uncropped cannabinoid receptors type 1 and 2 immunoblots. Uncropped images of immunoblots presented in Figure 3.

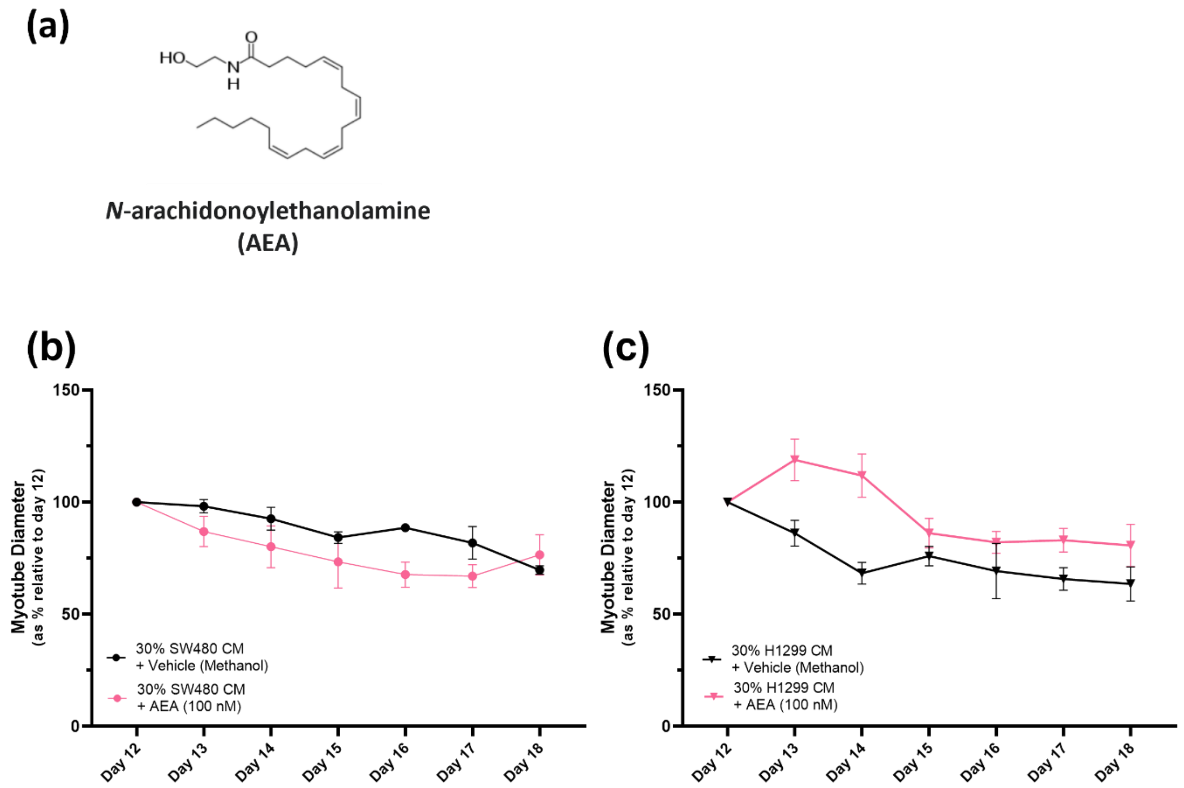

Figure S4. Effect of the endocannabinoid *N*-arachidonoylethanolamine (AEA) (CB_1R/partial CB_2R agonist) on cachexia model. (a) Structure *N*-arachidonoylethanolamine (AEA). **(b and c)** At day 12, cancer CM was introduced to induce cachexia; 30% SW480 CM/70% DM or 30% H1299 CM/70% DM and simultaneously; AEA [100 nM] or vehicle (Methanol [0.1%]) was added. Cells were monitored and myotube diameters were measured daily for 6 days. Data is presented as % relative to diameter of mature myotubes at day 12. All data is mean ± SEM of at least $n = 3$. AEA treatment was compared to its vehicle-treated counterpart under **(b)** 30% SW480 CM or **(c)** 30% H1299 CM conditions. AEA [100 nM] treatment did not affect the myotube degeneration caused by SW480 or H1299 CM induced cachexia.

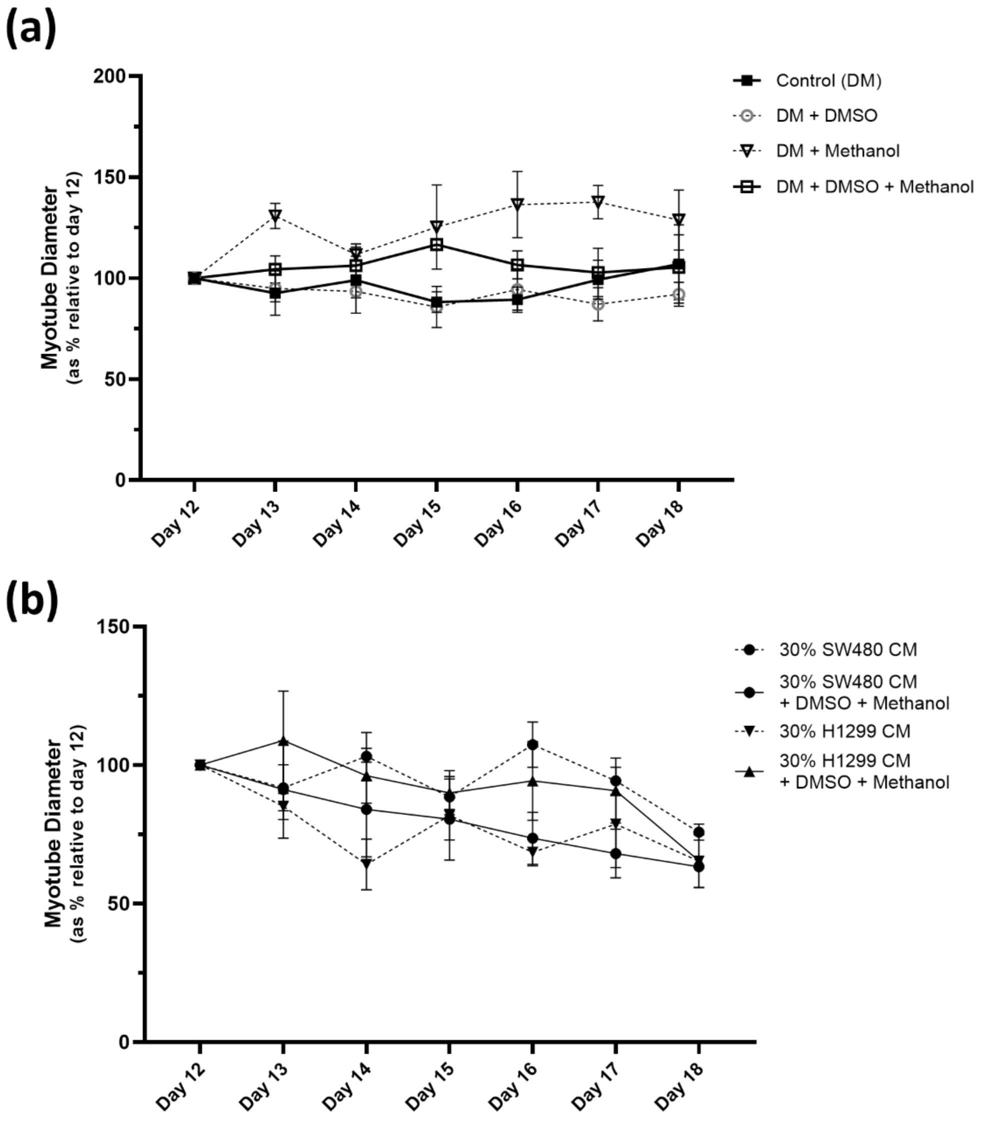

Figure S5. Effect of vehicles on myotubes. (a) Control (DM) myotubes are unaffected by treatment with DMSO [0.1%], methanol [0.1%] or both DMSO and methanol versus no vehicle treatment. **(b)** The myotube degeneration observed with 30% SW480 or 30% H1299 CM treatment is unaffected by the addition of DMSO [0.1%], methanol [0.1%] or both DMSO and methanol.

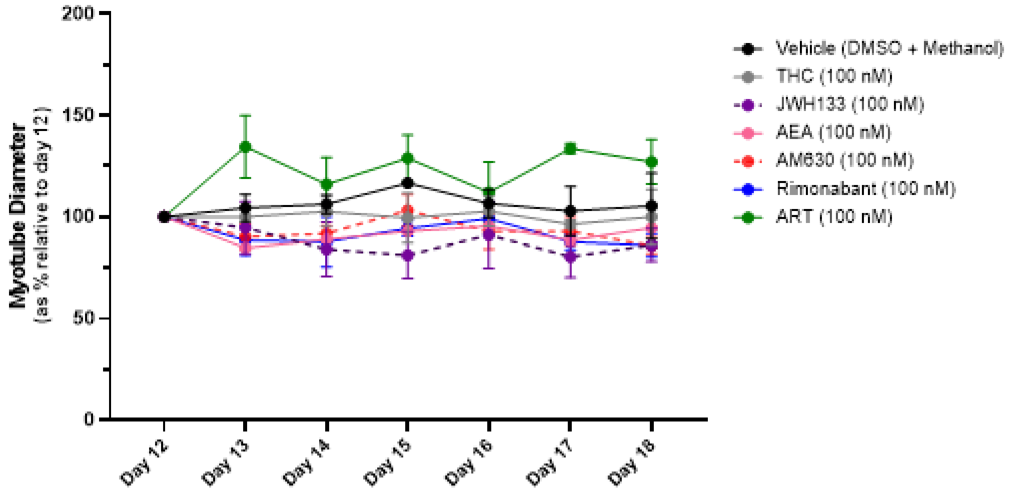

Figure S6. Effect of cannabinoid receptor agonists/antagonists on control (DM) myotubes. Control (DM) myotubes are unaffected by treatment with the cannabinoid receptor agonists/antagonists (THC, JWH133, AEA, Rimonabant, AM630 and ART27.13; all at [100 nM]) examined compared to vehicle control (DMSO [0.1%] and methanol [0.1%]).
